# Mandibular simple bone cysts: a rare case of bilateral occurrence

**DOI:** 10.1590/S1808-86942012000200022

**Published:** 2015-10-20

**Authors:** João Frank Carvalho Dantas de Oliveira, Danilo Baptista Martins Barbosa, Lucas Cavalieri Pereira, Marisa Aparecida Cabrini Gabrielli, Viviane Almeida Sarmento

**Affiliations:** a Doctoral degree in stomatology (Professor of stomatology); b Doctoral degree in stomatology (Professor of surgery, Dental School, UFPB); c Master's degree in oral and maxillofacial surgery (Oral and maxillofacial surgeon); d Doctoral degree in oral and maxillofacial surgery (Assistant professor, Department of Diagnosis and Surgery, UNESP, Araraquara, São Paulo, Brazil); e Doctoral degree in stomatology (Assistant professor, Department of Propedeutcs and Integrated Medicine, UFBA, Salvador, Bahia, Brazil)

**Keywords:** bone cysts, jaw cysts, nonodontogenic cysts

## INTRODUCION

The simple bone cyst is classified as an intraosseous bone pseudocyst; it is a pathologic cavity with no epithelial lining^1^, ^2^. Most cases are asymptomatic and are found in routine radiographs^1^, ^2^, ^3^.

The simple bone cyst comprises 1.25% of cysts of the jaw^1^, ^2^, ^3^. Most are unilocular^4^. Rarely more than one cyst may be found in a same patient – a few cases have been described^1^, ^3^, usually associated with cemento-osseous dysplasia^5^. This paper is a case report of a rare case of multiple simple bone cysts in the jaw of a patient with no associated cement-osseous lesions.

## CASE REPORT

A 22-year-old male patient was referred by an orthodontist because of asymptomatic radiolucent areas on routine radiographs.

The physical examination showed no bulging of the mandibular cortex, displacement, mobility, or loss of vitality of teeth adjacent to the pathologic areas. The patient reported no local trauma. Blood work-up (serum calcium, phosphorous, alkaline phosphatase, and PTH) were within normal limits.

**Figure 1:**
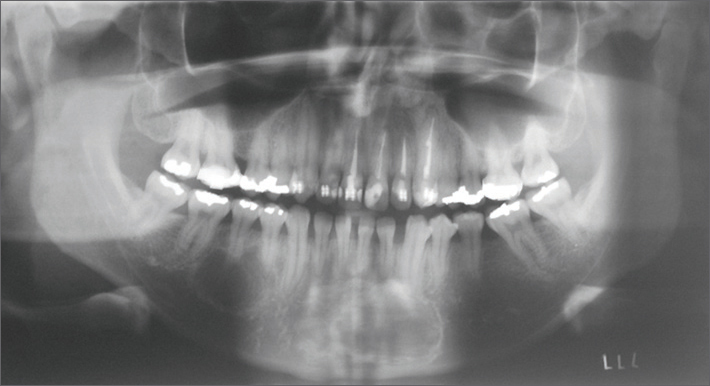
Panoramic preoperative radiograph showing radiolucent areas and projections between the apexes of adjacent teeth to the lesion in the body of the mandible.

Radiographic findings consisted of two unilocular radiolucent areas with a sclerotic halo that did not cause absorption of tooth roots, and that were located in the left parasymphyseal region in the mandible (measuring about 2.5 cm) and the right body of the mandible (diameter of about 2 cm) (Figure 1).

The first clinical and image diagnosis was keratocystic odontogenic tumor. The proposed treatment was enucleation of the lesions and curettage. The patient, however, did not visit the hospital on the scheduled day of surgery.

Three years later the patient returned for a reassessment. No changes were found in the physical examination compared to the previous evaluation. Panoramic radiographs showed that the two cysts had increased in size by about 1 cm each. There were projections between the apexes of adjacent teeth in the lesions located in the body of the mandible, which raised the possibility of simple bone cysts. Again, the patient was referred for surgery.

Surgery was done with local anesthesia. Needle aspiration of the cavities was negative for fluid. Corticotomy of the cysts revealed no signs of a cystic capsule or any other type of soft tissue within the bone cavity. The diagnosis of simple bone cyst was based on clinical, radiographic, and surgical features. The cavity borders were curetted.

Radiographs two years later showed that the radiolucent areas had regressed and that new bone had formed in the site; the cysts did not recur.

## DISCUSSION

Several radiolucent intraosseous lesions may arise in the jaws; most are asymptomatic and are discovered in routine radiographs^4^, ^6^ as in the present case. Most of these are unifocal lesions^6^. In the case above there were two radiolucent areas in the jaw. This situation generally occurs when there are endocrine or metabolic disorders such as hyperparathyroidism or, more commonly, in cases of multiple keratocystic odontogenic tumors, which are generally associated with the Gorlin-Goltz syndrome^6^. In the present case there were no metabolic disorders or manifestations of the Gorlin-Goltz syndrome.

It is rare for more than one simple bone cyst to occur in the same patient; such cases have been described in association with cement-osseous dysplasia^5^. In the study case there was no maxillary dysplasia. The diagnosis was only confirmed during surgery – a finding of empty bone cavities^4^, ^5^.

## FINAL COMMENTS

Cases of asymptomatic radiolucent lesions in the jaws, in which the clinical, laboratory or image diagnosis is not clear, require a biopsy because of the possibility of aggressive diseases being diagnosed erroneously as simple bone cysts. Computed tomography may be important for the diagnosis, as this method evaluates the content of lesions by Hounsfield density. The same applied to cases of multiple simple bone cysts, or cases in which there is an association with cement-osseous dysplasia, which suggests a more insidious condition. In such cases, long-term monitoring after curettage is needed so that recurrences be promptly diagnosed and treated.
